# Tubulin Cytoskeleton in Neurodegenerative Diseases–not Only Primary Tubulinopathies

**DOI:** 10.1007/s10571-022-01304-6

**Published:** 2022-11-09

**Authors:** Zuzanna Cyske, Lidia Gaffke, Karolina Pierzynowska, Grzegorz Węgrzyn

**Affiliations:** Department of Molecular Biology, Faculty of Biology, University of Gdansk, Wita Stwosza 59, 80-308 Gdansk, Poland

**Keywords:** Cytoskeleton, Microtubules, Neurodegenerative diseases, Tubulin

## Abstract

Neurodegenerative diseases represent a large group of disorders characterized by gradual loss of neurons and functions of the central nervous systems. Their course is usually severe, leading to high morbidity and subsequent inability of patients to independent functioning. Vast majority of neurodegenerative diseases is currently untreatable, and only some symptomatic drugs are available which efficacy is usually very limited. To develop novel therapies for this group of diseases, it is crucial to understand their pathogenesis and to recognize factors which can influence the disease course. One of cellular structures which dysfunction appears to be relatively poorly understood in the light of neurodegenerative diseases is tubulin cytoskeleton. On the other hand, its changes, both structural and functional, can considerably influence cell physiology, leading to pathological processes occurring also in neurons. In this review, we summarize and discuss dysfunctions of tubulin cytoskeleton in various neurodegenerative diseases different than primary tubulinopathies (caused by mutations in genes encoding the components of the tubulin cytoskeleton), especially Alzheimer’s disease, Parkinson’s disease, Huntington’s disease, amyotrophic lateral sclerosis, prion diseases, and neuronopathic mucopolysaccharidoses. It is also proposed that correction of these disorders might attenuate the progress of specific diseases, thus, finding newly recognized molecular targets for potential drugs might become possible.

## Introduction—Neurodegenerative Diseases

A progressive loss of neurons in the central nervous system (CNS) is characteristic for a group of several disorders called neurodegenerative diseases. Specific symptoms of particular diseases depend on the etiology, pathomechanism, and localization of pathological processes in the brain or other parts of CNS. The most frequent and commonly known neurodegenerative diseases are Alzheimer’s disease (AD), Parkinson’s disease (PD), Huntington’s disease (HD), and amyotrophic lateral sclerosis (ALS), though there are hundreds of others, and many of them are rare diseases (Dugger and Dickson 2017).

Neurodegenerative diseases can be either congenital or acquired (Dugger and Dickson 2017). Their primary mechanisms involve (i) accumulation of protein aggregates which impair functions of neurons (Shastry 2003), neuroinflammation (Chen et al. 2016), disorders in functions of mitochondria and other organelles (Giorgi et al. 2018), and demyelination (Lucchinetti et al. 2000). All of them lead to irreversible loss of neuronal functions and subsequent loss of neurons due to cell death (Dugger and Dickson 2017).

Searching for effective therapies for neurodegenerative diseases is undoubtedly necessary and urgent. This is, on the other hand, an extremely difficult task due to complex pathomechanisms of these diseases, their variabilities and different symptoms observed in patients. Moreover, ability of neurons to regenerate is extremely limited. Unfortunately, vast majority (if not all) of neurodegenerative diseases remain incurable (Pierzynowska et al. 2020).

## Cytoskeleton with Special Emphasis on Microtubules

Cytoskeleton is a network of interlining filamentous proteins occurring in cytoplasm. It was assumed that it occurs solely in eukaryotic proteins, however, some 30 years ago, protein motifs homologous to actin (Bork et al. 1992) and both subunits of tubulin (*α*-tubulin and *β*-tubulin) (de Boer et al. 1992; RayChaudhuri and Park 1992; Mukherjee et al. 1993; Yutin and Koonin 2012) were discovered in bacteria and archea. Nevertheless, it is unlikely that the cytoskeleton of eukaryotic cells derives from prokaryotes, as detailed analyses indicated significant functional differences between components of cytoskeletal proteins occurring in eukaryotes and their bacterial structural analogs (Vesteg and Krajcovic 2008). In fact, actin and tubulin, major proteins of the cytoskeleton in eukaryotic cells, are absent in prokaryotes, however, there are their homologues, MreB (the actin homologue) and FtsZ (the tubulin homologue) which can be found in bacterial and archeal cells (Carballido-López and Errington 2003). The MreB protein is involved in the regulation of the shape of bacterial cell and in partition of plasmid molecules into newly formed cells, while FtsZ is crucial for proper cell division (Amos et al. 2004).

The primary function of the cytoskeletal filaments is their action as “linear machines”, i.e., cytomotoric abilities (Löwe and Amos 2009). In eukaryotes, such functions are evidently expanded due to dynamic polymerization and depolymerization of microtubules (McIntosh et al. 2010) as well as appearance of additional “machines”, like dynein, myosin, and kinesin (Kull et al. 1996; Roberts et al. 2013; Cianfrocco et al. 2015) which use the energy from ATP hydrolysis (Wickstead and Gull 2011). In fact, dynein and kinesin are also involved in dynamic microtubule polymerization (Hunter and Wordeman 2000).

In eukaryotes, cytoskeleton is the cellular scaffold, ensuring the spatial organization of the cell. Moreover, it facilitates contact of the interior of the cell with the environment, movement of organelles, and cell division. Such functions are possible because cytoskeleton connects activities of various organelles (Wickstead and Gull 2011). Another classification of cytoskeleton’s functions indicates three categories: (i) spatial organization of the cell, (ii) contact of the cell interior with the environment, and (iii) movement and cell shape modulation (Fletcher and Mullins 2010). Structurally, cytoskeleton consists of actin filaments (microfilaments), microtubules, and intermediate filaments, playing different roles in the cell physiology. Nevertheless, all these polymers are organized in a network which accommodates cells to various environmental and physiological conditions (Fletcher and Mullins 2010). Organization of this network is controlled by various proteins, including nucleation-promoting factors, capping proteins, polymerizing agents, depolymerizing factors and severing factors, and stabilizing and crosslinking proteins. The major differences between three main components of the cytoskeleton are stiffness of the filaments, dynamics of their accumulation, and types of molecular machines they cooperate with (Fletcher and Mullins 2010). In this review, we will focus on microtubules and their dysfunctions in neurodegenerative diseases, while other major cytoskeleton elements were described elsewhere (for historical and current aspects of these structures, see Flaherty et al. (1991), Mitchison (1992), Lodish et al. (2000), van den Ent et al. (2001), and Esue et al. (2005) for microfilaments, and Erber et al. (1984) and Vermeire et al. (2021) for intermediate filaments).

Microtubules are major components of the cytoskeleton (Dustin 1984; Bray 2000). They consist of tubulin, a heterodimer of two subunits, *α*-tubulin and *β*-tubulin, which interact forming structures of hollow cylinders (Cooper 2000). These heterodimers are connected in the “head-to-tail” manner, building linear polymers called protofilaments (Horio and Murata 2014). Microtubules are the most complex structures among the three polymers forming the cytoskeleton. They are responsible for modulation of the cell shape, and they are involved in intracellular signal transduction and organization of the cytoplasm (Alfaro-Aco and Petry 2015).

Dynamic instability, the phenomenon discovered almost 40 years ago (Mitchison and Kirschner 1984a, b), is a crucial feature of the tubulin cytoskeleton. Due to alternating polymerization and depolymerization of microtubules, which leads to their stabilization and destabilization, respectively, intracellular movement of organelles is possible which depends on changing environmental conditions (Fletcher and Mullins 2010). The polymerization and depolymerization proceeds through addition or removal of one of subunits (*α*-tubulin or *β*-tubulin) from corresponding end of the microtubule. The “minus” end, with the terminal *α*-tubulin, is formed significantly slower than the “plus” end, with the terminal *β*-tubulin (Cote and Borisy 1981; Mitchison 1993).

The rate of growth and decay of microtubules depends on the concentrations of tubulin subunits (Honda et al. 2017). Under conditions supporting polymerization and formation of microtubules, the growth rate depends on levels of both tubulin subunits in cells. However, the rate of depolymerization is usually several times higher, and it is independent on tubulin subunits’ concentrations (Horio and Hotani 1986; Kirschner and Mitchison 1986; Walker et al. 1988; Alfaro-Aco and Petry 2015). Molecular mechanism of microtubule polymerization appears complicated, including permanent and apparently random changes of protofilaments’ ends at intervals between relatively slow growth and rapid shrinkage of microtubules (Kerssemakers et al. 2006; Schek et al. 2007; Gardner et al. 2008). It is assumed that stability of microtubules depend mainly on the stability of the cap, i.e., the GTP-tubulin form at the ends of these structures, and dynamics of the GTP-tubulin vs. GDP-tubulin conversion (Carlier et al. 1987; O'Brien et al. 1987; Bayley et al. 1990; Drechsel and Kirschner 1994; Caplow and Shanks 1996; Schek et al. 2007; Dimitrov et al. 2008).

Microtubules can interact with other proteins. Some of these proteins can cleave microtubules, while others stimulate depolymerization and enhance its rate. There is a specific kind of proteins which cooperate with microtubules and increase their stability, called microtubule-associated proteins (MAP) (Hamada 2007; Gardiner 2013). They stabilize microtubules through protecting from disaggregation of tubulin subunits and promoting addition of new subunits and the growth (Horio and Murata 2014). There are various MAPs which functions vary depending on the cell type. Among the best studied MAPs, there are MAP-1, MAP-2, and tau, occurring in neuronal cells, and MAP-4 in non-neuronal cells of vertebrates. In neurons, MAPs localize in both dendrites and axons, however, tau protein predominates in axons while MAP-2 occurs mostly in dendrites (Cooper 2000).

## Tubulin Cytoskeleton Dysfunctions in Neurodegenerative Diseases

Dysfunctions of the cytoskeleton are often described as characteristic features of neurodegenerative diseases. Here, we focused on tubulin cytoskeleton dysfunctions in various kinds of such disorders. In fact, primary neurodegenerative tubulinopathies, caused mainly by mutations in genes encoding the components of the tubulin cytoskeleton, especially in *TUBA4A*, *TUBB2A*, and *TUBB3* genes, have been excellently reviewed and comprehensively discussed recently (Sferra et al. 2020). Therefore, in this review article, we aimed to present and discuss problems which appear in the tubulin cytoskeleton as secondary effects of the pathological mechanisms which nevertheless play considerable roles in development of cellular disorders leading to impairment of neuronal functions and facilitating neurodegeneration. Alzheimer’s disease, Parkinson’s disease, other amyloidopathies and tauopathies, and remaining diseases in which secondary changes in the tubulin cytoskeleton contribute to neurodegeneration will be discussed.

### Alzheimer’s Disease

At the biochemical level, Alzheimer’s disease is characterized by accumulation of insoluble forms of beta-amyloid (Aβ) and hyperphosphorylated tau protein (P-tau). There are ongoing discussions and debates on specific mechanisms of P-tau and Aβ pathogenicity, and various hypotheses have been presented, like neuronal cell death induced by abnormal P-tau fibrils in association with neuronal lesions and activation of glial cells caused by Aβ (Iwatsubo 2022), or neuroinflammation triggered by microglia metabolic reprogramming due to accumulation of Aβ and P-tau and their propagation to adjacent microglia through exosomes (Zhao et al. 2022). Although it was supposed previously that Aβ and P-tau act independently, results of recent studies suggested that these two factors can interact and such interactions can contribute significantly to development of pathological changes, as summarized and discussed recently in detail by Busche and Hyman (2020). The chronology of Alzheimer’s disease development is also not fully understood. Nevertheless, recently Parodi-Rullán et al. (2021) summarized our knowledge in this matter, and proposed the scheme according to which the disease in initiated by Aβ, P-tau, and vascular risk factors that cause dysfunctions of mitochondria, leading to production of reactive oxygen species in cerebral endothelial cells. This can activate vascular inflammation that stimulates microglia and astrocytes, triggering the inflammatory cascade. As the inflammation becomes widespread, the blood–brain-barrier function is impaired, leading to neuroinflammation and further to neurodegeneration. In this light, it is important to note that recent review of medical data and analyses and experimental results led to the conclusion that the primary deleterious effects of P-tau are related to mitochondrial dysfunctions (Epremyan et al. 2022).

Despite large number of studies on the pathomechanisms of Alzheimer’s disease, summarized briefly in the preceding paragraph, it is unclear how disturbances in the structure and organization of microtubules and microtubule-associated proteins contribute to the pathogenesis of this disease. It appears that dysfunctions of the tau protein might play the crucial role there. This protein is normally associated with microtubules and promotes their assembly, as well as stabilizes these structures (physiological roles of tau have been reviewed recently by Liang et al. 2022). Due to alternative splicing of mRNA of the tau-encoding gene (the *MAPT* gene), there are 6 isoforms of the tau protein which differ in the length, ranging from 352 to 441 amino acid residues. These isoforms are classified as 3R (including 0N3R, 1N3R, and 2N3R isoforms) and 4R (including 0N4R, 1N4R, and 2N4R isoforms) groups, and dysfunctions of each isoform, caused by specific mutations in *MAPT*, overexpression of this gene, and/or aberrant posttranslational modifications of the gene product, cause different neurological disorders, as summarized and discussed thoroughly by Hernández et al. (2022) and Zhang et al. (2022). There are several dozens of phosphorylation sites in tau which are important in modulation of functions of this protein (as reviewed recently by Wegmann et al. 2021). In Alzheimer disease, it was indicated that pathological changes are related mainly to the hyperphosphorylation of the tau protein at Ser199 and Ser262, as well as Thr212 and Thr231 (Alonso et al. 1996, 2018), though it must be stressed that pathological changes found in this disease are not restricted to these phosphorylation sites. This was discussed recently by Carroll et al. (2021) who indicated that hyperphosphorylation at Thr181, Thr231, and Ser396 cause mislocalization of the tau protein, its oligomerization, and subsequent intracellular accumulation with possible extracellular export. In the course of progression of Alzheimer disease, intraneuronal neurofibrillary tangles, composed of P-tau, accumulate sequentially in various regions of the brain, starting from entorhinal and peripheral cortex, then expanding to the hippocampus (especially the CA1 region) and the limbic regions, further to amygdala, thalamus, claustrum, the isocortical areas, and finishing in primary sensory, motor, and visual nexuses (for a recent review, see Carroll et al. 2021).

To investigate a role for P-tau in Alzheimer’s disease, a mouse model has been constructed using doxycycline to hyperphosphorylate the tau protein (Di et al. 2016). Studies have shown that in mice with induced P-tau overproduction, the number of neurons in the hippocampus decreased. The initial stage of cytoskeletal impairment is most likely the disconnection of the normal form of the tau protein from the microtubules, which increases its instability (Di et al. 2016).

Paeoniflorin is a glycoside derived from a peony plant (*Paeonia lactiflora*) which is used in traditional Chinese medicine, and it was reported to have a beneficial effect on neurodegenerative diseases (Wang et al. 2021). In fact, transmission electron microscopy studies showed that paeoniflorin may reduce the number of autophagosomes and stabilize the microtubule structure (Chen et al. 2017). However, the effect of paeoniflorin on tauopathies remains inconclusive. It was found that paeoniflorin protects SH-SY5Y cells from the effects of okadaic acid, which lead to the phosphorylation of the tau protein, interfering with calpain/Akt/GSK-3*β* pathways, in which autophagy may be involved (Ma et al. 2018). Other studies suggested that Al^3+^, Fe^3+^, and Zn^2+^ ions may disrupt microtubule formation in rat brains (Shevtsov et al. 2016). Microtubules were formed only in the presence of Al^3+^ and Fe^3+^ ions. Therefore, Zn^2+^ ions are most likely not involved in production of the pathological form of microtubules in Alzheimer's disease. It was proposed that Al^3+^ ions are the most likely cause of the disease due to their highest concentration in the brain in Alzheimer’s patients (Shevtsov et al. 2016). However, despite an evident relationship between naturally occurring metals and the development or progression of Alzheimer’s disease, there is not yet evidence whether such relationship actually causes this disease (Shaw 2018). Co-localization of Al^3+^ with the phosphorylated tau protein has been demonstrated in the brains of patients with familial form of Alzheimer’s disease (Mold et al. 2021). These results led to the proposal that Al^3+^ contributes to the neuropathology of this disease and that potential reduction of levels of this metal ions might have a therapeutic benefit (Mold et al. 2021).

Using the transgenic *Drosophila melanogaster* model of co-production of the human tau and Aβ proteins, it was proved that tau phosphorylation at Ser262/356 stabilized free tau in the early phase of abnormal tau formation, leading to neurodegeneration. Aβ increased the level of tau detached from microtubules, regardless of the state of phosphorylation. Misplaced tau proteins, especially less phosphorylated forms, were stabilized by phosphorylation at Ser262/356 by PAR-1/MARK (Ando et al. 2016). It was also suggested that abnormalities in the localization of the tau protein might be correlated with the loss of microtubules and further consequences such as loss of mature spines, loss of synaptic activity, and abnormal mitochondrial localization (Zempel and Mandelkow 2015). Tubulin tyrosine ligase like 6 (the TTLL6 protein) stimulates microtubule polyglutamylation which induces spastin-mediated microtubule breakdown. The loss of microtubules prevents cells from maintaining mitochondrial transport, which in turn results in synaptic dysfunction and loss of mature spines (Zempel and Mandelkow 2015).

Abnormal post-translational modifications are likely involved in the pathological process in Alzheimer’s patients. It would be a great achievement in understanding the role of tau truncation to identify the exact cleavage sites for truncated tau fragments, especially those truncated at the *N*-terminus, which are less understood than those truncated at the *C*-terminus. It was possible to identify new forms of the tau protein with a truncated N-terminus. The studies with a cellular model, conducted to analyze the two *N*-terminally shortened forms of tau, starting with residues Met11 and Gln124, were described (Derisbourg et al. 2015). It was demonstrated that the Gln124-tau fragment has a stronger affinity for microtubule binding and stabilization, which indicates that the *N*-terminal domain of tau may play a key role in microtubule stabilization. In vitro and in vivo studies have shown that histone deacetylase 6 (the HDAC6 protein) interacts with tau. This interaction is mediated by the microtubule binding domain on the tau protein and the Ser/Glu tetradecapeptide domain on HDAC6. Treatment with tubacin, a selective inhibitor of HDAC6 tubulin deacetylation activity, did not cause disturbances in the HDAC6-tau interaction (Ding et al. 2018). In addition, it was observed that HDAC6 protein levels were significantly elevated in brains of individuals with Alzheimer’s disease (Ding et al. 2018). Therefore, one might propose HDAC6 as a tau-interacting protein and as a potential modulator of tau phosphorylation and accumulation. On the other hand, mice transgenic for tau and devoid of HDAC6 revealed increased tau pathology, accelerated cognitive decline and shortened life span (Trzeciakiewicz et al. 2020). Thus, there are contradictory data which did not allow to conclude whether a decrease or increase of HDAC6 level or activity might be beneficial for patients.

A comprehensive identification of harmful post-translational modifications was performed on the basis of mass spectrometry data from soluble (normal) and insoluble protein fractions, as well as total extracts from control, for selected proteins (Aβ, tau, apolipoprotein (apo) E, glial fibrillar acid protein (GFAP), tubulin a-III and tubulin b-III) (Boutte et al. 2006). Methionine sulfoxide at Aβ and numerous tau protein phosphorylation sites in the insoluble proteins characteristic for Alzheimer’s disease were identified, while no post-translational modifications were enriched with ApoE or GFAP (Boutte et al. 2006).

Recent studies have shown that silencing of chaperone E, which is necessary for proper tubulin formation, can cause tau protein neurotoxicity (Fujiwara et al. 2020). Consequently, one might propose that disrupted tubulin homeostasis can result in abnormal tau formation and ultimately cause a tauopathy. To test this hypothesis, a knockdown system was developed using miRNA for tubulin-specific chaperone E gene (*Tbce*). Knockdown of the *Tbce* expression in primary neuronal cultured cells induced an increase in tubulin levels. The accumulated tubulin was neither acetylated nor incorporated into microtubules, indicating that the molecules were functionally inert (Fujiwara et al. 2020). These results indicated that the tubulin-specific chaperone E induces not only the reduction of correctly folded tubulins, which are capable of forming microtubules, but also the accumulation of phosphorylated tau in the cell body of mammalian neurons. In fact, earlier studies showed that P-tau prevents proper microtubule formation in patients suffering from Alzheimer’s disease by trapping the normal form of the tau protein (N-tau) and microtubule-associated proteins MAP1 and MAP2; interestingly, dissociation of hyperphosphorylated N-tau fibers by ultrasound restored their ability to bind N-tau (Alonso et al. 1996, 1997, 2006). These findings suggested that P-tau is most likely responsible for the breakdown of microtubules in neurons in Alzheimer’s patients.

Undoubtedly, post-translational modifications of tubulin play crucial roles in the regulation of neuronal microtubule cytoskeleton functions. Among them, tyrosination, acetylation, polyglutamylation, and SUMOylation appear the most important ones (Feng et al. 2021; Moutin et al. 2021). Disturbances in these protein modification processes were reported in Alzheimer’s disease and other neurodegenerative disorders (Santiago-Mujika et al. 2021). Recent studies demonstrated that disturbed regulation of polyglutamylation might be especially disturbing for microtubule structure and functions, leading to neurodegeneration processes (Bodakuntla et al. 2021a). Although polyglutamylation appeared to be required for microtubule growth (Baird and Bennett 2013), it was demonstrated that abnormally high level of this modification, called hyperglutamylation, caused by impairment of the deglutamylation process, could lead to neuronal death (Magiera et al. 2018; Shashi et al. 2018). Interestingly, results of the latter studies suggested that the mechanism of such neurodegeneration might involve defects in axonal transport mediated by microtubules (Akhmanova and Hoogenraad 2018). Subsequent experiments demonstrated that dysfunction of the major deglutamylase, the CCP1 (also called AGTPBP1) protein, resulted in hyperglutamylation-dependent reduction in motility of mitochondria, lysosomes, endosomes and other intracellular vesicles (Bodakuntla et al. 2020). Therefore, one could propose that hyperglutamylation-caused neurodegeneration depends on the disturbed intracellular trafficking and transportation. Indeed, such a suggestion has been corroborated recently by Lopes et al. (2020). Moreover, enzymes catalyzing specific polyglutamylation of α-tubulin and *β*-tubulin, TTLL1 and TTLL7, respectively, were identified, and the former one has been recognized as a factor responsible for hyperglutamylation that resulted in changes in motility of mitochondria in neurons (Bodakuntla et al. 2021b). Hence, it was proposed that enzymes involved in the control of polyglutamylation of tubulin might be considered as potential targets for novel anti-neurodegenerative drugs (Ramadan et al. 2021; Santiago-Mujika et al. 2021). Perhaps such a therapeutic approach should concern especially the process of α-tubulin polyglutamylation.

**Fig. 1:**
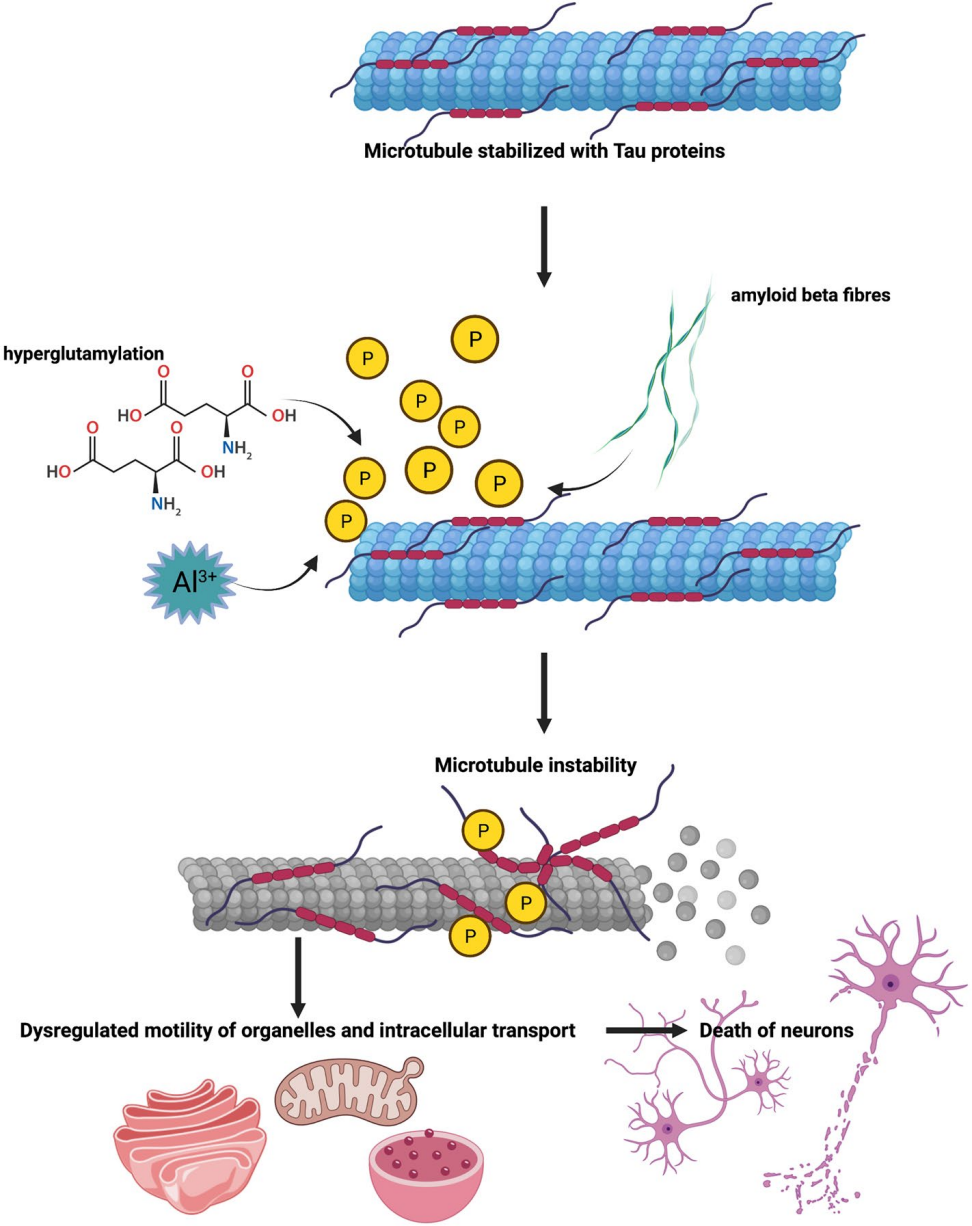
Major microtubule-related pathomechanisms of Alzheimer’s disease. Microtubules are normally stabilized by the tau protein. However, various factors/processes, like *β* amyloid, hyperphosphorylated tau protein (*P*-tau), elevated levels of Al^3+^ ions, and hyperglutamylation, lead to destabilization of microtubules in neurons which causes defects in motility of organelles and intracellular transport. These are followed by neuron death. Figures were created using BioRender.com

**Fig. 2:**
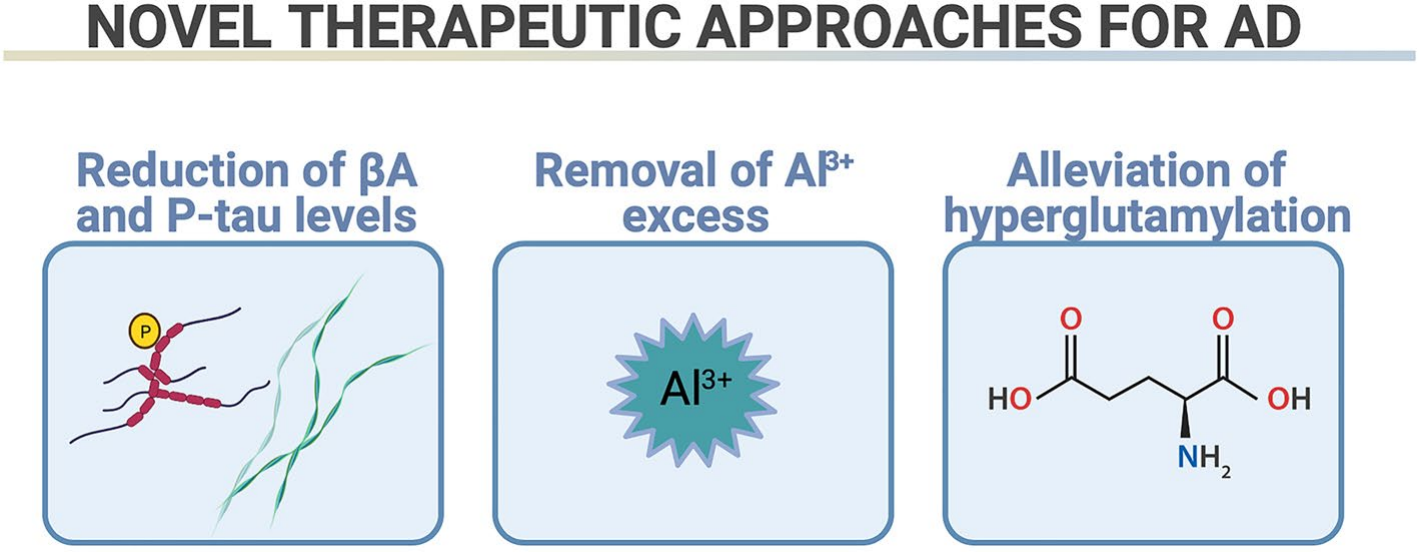
Novel therapeutic approaches for Alzheimer’s disease (AD). Decreasing levels of β amyloid (Aβ) and hyperphosphorylated tau protein (P-tau) (for example by autophagy stimulation), removal of excess of Al^3+^ ions, and alleviation of hyperglutamylation might protect microtubules and prevent neurodegeneration. Figures were created using BioRender.com

In summary, microtubule disorders are evidently connected to Alzheimer’s disease. However, dysfunctions of microtubules are complex and can be caused by various agents. Major microtubule-related pathomechanisms of Alzheimer’s disease which lead to death of neurons are summarized in Fig. 1. Definitely, P-tau contributes significantly to microtubule breakdown, while disrupted tubulin homeostasis facilitates tauopathy, leading to specific positive feedback and a spiral of pathogenicity. Although most therapeutic approaches in treatment of Alzheimer’s disease have been focused on elimination of Aβ, it appears that even very effective methods of disruption of aggregates of this protein are insufficient for clinical improvement of patients (Kim et al. 2022). One possible reason might be still ongoing tauopathy and resultant secondary tubulinopathy which in turn accelerates tauopathy. Therefore, we suggest that disruption of such a pathogenicity spiral might be beneficial or even necessary to achieve considerable therapeutic effects. Indeed, a therapy which simultaneously reduced levels of both Aβ and P-tau, due to stimulation of autophagy, resulted in correction of the disease symptoms in the rat model of the sporadic form of Alzheimer’s disease (Pierzynowska et al. 2019a). It is possible that such treatment eliminated both senile plaques and the complex microtubule-P-tau pathogenicity, allowing to normalize functions of neurons and the whole brain. On the other hand, one should remember about contribution of Al^3+^ ions to the pathogenic mechanisms of the disease, connected to microtubules, pointing to the necessity of removal of an excess of such ions if their accumulation occurs. Moreover, any disruptions of the processes controlling polyglutamylation of tubulin, and especially α-tubulin, may lead to disturbed regulation of mobility of organelles, impaired intracellular transport, and subsequent neurodegeneration. Thus, in patients suffering from Alzheimer’s disease in which the pathomechanisms are definitely more complex than in any animal model, where the disease is caused or induced by a single factor or agent, one should consider treatment procedures focused on many, rather than one, targets. Emerging therapeutical approaches for Alzheimer’s disease, proposed based on recent discoveries and related to microtubule disorders, are shown schematically in Fig. 2.

### Parkinson’s Disease and Remaining Tauopathies

In this sub-section we will summarize defects of tubulin cytoskeleton in Parkinson’s disease and other tauopathies. However, some aspects of Alzheimer’s disease (that is also a kind of tauopathy) will also be pointed which have connections to other tauopathies (despite presentation of Alzheimer’s disease in more detail in the preceding sub-section) to avoid artificial dividing of the problems when discussed mechanisms required for consideration of a broader spectrum of diseases.

#### Composition of Lewy Bodies and Other Abnormalities

Neurodegenerative diseases are often associated with the appearance of fibrous inclusions at later age, and these characteristic structures often separate from the cytoskeleton. As early as in 1988, an attempt was made to characterize the cytoskeletal compartment in Lewy bodies (LB), one of the markers of Parkinson's disease (Galloway et al. 1988). A set of antibodies specific for neurofilaments, tubulin, microtubule-associated proteins (tau, MAP1 and MAP2), and Alzheimer's neurofibrillary tangles (NFTs) was used. All microtubule-associated antibodies were used and some of those acting against NFTs recognized Lewy bodies. However, two of the latter antibodies also reacted with ubiquitin (Galloway et al. 1988). These results suggested that the presence of tau, the key component of NFT, may not be a prerequisite for the formation of abnormal filaments. Therefore, apparently Lewy bodies, like other abnormal neuronal filaments, differ in composition. In another study, NFT-like β-tubulin structures were found to be immunoreactive in Alzheimer's disease, more commonly, in Guam ALS/parkinsonism-dementia and in Down syndrome. The β-tubulin structures were intracellular and appeared early in the tangle development (Schwab and McGeer 1998). This provided evidence indicating the involvement of tubulin in NFT formation.

In primary cultures, aggregation of gamma-tubulin occurred under the influence of rotenone. Destabilization of microtubules, disruption of mitotic spindles and changes in the Golgi apparatus were observed. These results indicated that aggregation in the centrosome promotes the formation of inclusions, and the compound itself affects the structure and function of the centrosome, leading to disruption of the cytoskeleton, Golgi apparatus and ultimately cell disintegration (Diaz-Corrales et al. 2005). Puig et al. (2005) performed experiments confirming that high β II-tubulin content is a contributing factor to the formation of abnormal hyperphosphorylated tau aggregates.

Due to the composition of Lewy bodies and the appearance of specific inclusions as a result of proteasome inhibition or generation of misfolded proteins in dopaminergic neurons, it was indicated that their formation is a response to an increase in the level of abnormal proteins in neurons (McNaught et al. 2002). There is a potential effect of aggregated a synuclein on the microtubule network. A model with overexpression of the protein was used and indicated that the protein was specific, as aggregation of α synuclein and tubulin, but not actin, was observed. Thus, the microtubules constitute a potential target for α synuclein and alterations within them can lead to disorders of neuronal structures and functions (Lee et al. 2006). Abnormalities of the cytoskeleton in the brain of people with neurodegenerative diseases have been widely described, but an attempt was made to determine the stoichiometric ratios of the individual components. It was indicated that the disassembly of the cytoskeleton observed in patients with Down syndrome, Alzheimer's disease or Pick’s disease is associated with a reduced number of individual components, and thus it is a consequence of the disruption of the stoichiometric proportions of individual proteins in the brain (Pollak et al. 2003).

#### Tau Studies

To investigate how mutation-caused changes in the tau protein promote neurodegeneration in frontotemporal dementia and parkinsonism associated with chromosome 17, different variants of transgenic mice were created. Changes in the number and morphology of lysosomes similar to those found in Alzheimer’s disease were noted. These results indicated that lysosomal abnormalities may be the cause of neurodegeneration (Lim et al. 2001). The overproduction of the longest human tau isoform in primary cultures of rat astrocytes was analyzed. The results suggested that reduced stability of detyrosinated microtubules results from aggregation of the tau protein. This leads to further dysfunction and death of astrocytes in tauopathies (Yoshiyama et al. 2003; Kahlson et al. 2016). To test effects of the tau protein on membrane elements, wild-type protein and several truncated variants were overproduced in neuroblastoma cells. Tau-induced ring microtubule bundles were stable structures, resistant to nocodazole with extensive tubulin acetylation. The results indicated that it is the mechanical strength of the resulting structures that may contribute to the fragmentation of the Golgi apparatus (Rodríguez-Cruz et al. 2018).

How microtubule loss in tauopathies is related to disease pathogenesis is still unclear. The theory of the importance of "free tau" in the development of tauopathy was advanced by Miyasaka et al. (2018) based on studies on transgenic models of *Caenorhabditis elegans*. They pointed to the cytotoxic potential of the protein associated with microtubule-binding repeats. At older age, as a result of the exposure of these repeats, it behaves as a toxic agent or promotes its own aggregation (Miyasaka et al. 2018). Recent data indicated that decreased tubulin protein levels contribute to the neurotoxicity of the tau protein. A chaperone E knockout system of tubulin, which is involved in tubulin synthesis, has been developed. In primary cultures of mouse neurons, there is a reduction of properly organized tubulin and accumulation of phosphorylated tau (Fujiwara et al. 2020). These results indicate an important involvement of disruption of tubulin biosynthesis in the accumulation of toxic tau protein.

Disturbed carbohydrate metabolism can lead to an increased risk of developing neurological diseases in patients with diabetes. Toxic glycation products (TAGE, AGE) contribute to the phosphorylation of the tau protein. The effect of glyceraldehyde (GA) on SH-SY5Y neuroblastoma cells was investigated to find that β-tubulin was identified as one of the target proteins for TAGE. There was abnormal aggregation of this protein under the influence of GA. Importantly, AGEs from glucose did not have this effect. It was proposed that elucidation of the mechanism of GA action in neurodegeneration may contribute to the development of new neuroprotective therapies (Nasu et al. 2020). In another study on the role of tau, the effects of its full length (Tau 441) and a shortened, pathologic form (Tau 421) on cytoskeleton and plasma membrane functions were evaluated. Abnormal microtubule binding was observed in C6 glial cells. Moreover, under the influence of both protein variants, there was a disruption of the plasma membrane which was associated with a change in the distribution of filamentous actin that did not occur after treatment with an inhibitor of the Rho protein kinase signaling pathway. These effects were reversed when depolymerizing compounds were applied. Taken together, these facts might indicate a newly discovered mechanism of tau protein toxicity in Alzheimer’s disease and other tauopathies (Torres-Cruz et al. 2016). Irreversible loss of taxol-stimulated tubulin polymerization was demonstrated in samples from patients with late-onset Alzheimer’s disease, patients with *PSEN2* and *FTDP-17* mutations, while it did not occur in subjects with mild cognitive impairment or controls. It seems that modification of tubulin may lead to intermediate or late stages of the course of both sporadic and inherited Alzheimer’s disease or frontotemporal dementia with Parkinsonism linked to chromosome 17 (also known as frontotemporal dementia 17 or FTDP-17) (Boutté et al. 2005). Using a proteomic approach, several N-terminally-truncated tau variants were identified in samples from human brain. Cellular studies indicated the importance of the N-terminal domain of the protein in microtubule stabilization. Data on the role of tau, spastin, and the long chain polyglutamylation enzyme in Alzheimer’s disease and hereditary spastic paraplegia, along with therapeutic options were summarized previously in a comprehensive review paper (Zempel and Mandelkow 2015).

#### Frontotemporal Dementia with Parkinsonism Linked to Chromosome 17, Other Tauopathies and Therapeutic Perspective

Frontotemporal dementia with Parkinsonism linked to chromosome 17 (FTDP-17) is a disease inherited in an autosomal dominant manner. Tau-encoding gene mutations were assessed by Southern blotting, as well as splicing abnormalities in a Swedish family. The presence of other mutations in the 17q21 region, like in a gene coding for gamma-tubulin, was also assessed. No pathological changes were observed, so further analyses are mandatory (Froelich Fabre et al. 2003). Another study examined the effects of four mutations (p.G272V, p.P301L, p.V337M, and p.R406W) on microtubules. It turned out that a given mutation affected them in a site- and isoform-dependent manner. Tau R406W did not form filament structures in confocal imaging, which may indicate a distinct carboxyl region in microtubule assembly. In addition, the mutant form of the protein was not phosphorylated. Thus, the experimental results indicated a reason for the diverse clinical pathologies of FTDP-17 and related tauopathies (Sahara et al. 2000). One concept is that mutant tau complexes are formed with wild-type tau that have altered biological and/or biophysical properties that promote the emergence of the FTDP-17 phenotype (Makrides et al. 2003).

The β IV-tubulin is one of the targets for oxidative damage. Biochemical changes and effects of oxidative stress were found in the frontal cortex of patients suffering from Parkinson’s disease associated with the p.G2019S mutation in the *LRRK2* gene, encoding leucine-rich repeat kinase 2. The changes were similar to those occurring in sporadic Parkinson’s disease without cognitive impairment (Gomez and Ferrer 2010). Increased Parkinson’s disease risk characterized mice lacking the pituitary adenylate cyclase-activating polypeptide (PACAP). It appears that ATP synthase and β IIA-tubulin are up-regulated there, whereas levels of various enzymes were down-regulated which may result in increased susceptibility of PACAP-deficient animals (Maasz et al. 2014).

Interestingly, abnormalities in the shape or distribution of neurons are observed in brain areas of patients with schizophrenia. An increase in levels of unphosphorylated MAP2 and MAP1B is thought to produce these abnormalities. NFS are more frequently observed in patients undergoing antipsychotic pharmacotherapy. In fact, numerous data indicate similarity of cytoskeletal changes in neurons in neurodegenerative and psychiatric diseases. Importantly, such disorders may be therapeutic targets. For example, there are data indicating the efficacy of melatonin, which affects cytoskeletal remodeling (Benitez-King et al. 2004). Another therapeutic approach targeting microtubules is to use stabilizing agents and attempt to improve axonal transport function and neuronal function. However, it is worth to note that there are many challenges associated with using this strategy (Brunden et al. 2017).

A specific mutation designed to mimic acetylated microtubules was created using the CRISPR/Cas9 technique in *Drosophila*. It appears that tubulin acetylation rescues tau-induced defects. In addition, late administration of HDAC6 inhibitors also led to improvement (Mao et al. 2017). Such results indicate that increasing microtubule acetylation through genetic manipulation or drugs may be used as a potential strategy for intervention in tauopathies. Interesting research was conducted through treatment SH-SY5Y cells with okadaic acid for 8 h to induce tau phosphorylation. Next, effects of paeoniflorin on the okadaic acid-induced cytoskeletal associated changes were examined. It appears that paeoniflorin attenuates the stress response on microtubules via the calpain/Akt/GSK-3*β* pathway (Ma et al. 2018). A large therapeutic potential seems to be characterized by compounds modifying the tau protein (Soeda and Takashima 2020), which is being intensively studied, although clinical evidence is not yet available. Recently, mutations in genes coding for enzymes involved in microtubule modifications were discovered in patients with early neurodegeneration. There are emerging indications that targeting these enzymes may provide an opportunity in the treatment of neurodegenerative diseases (Rogowski et al. 2021). Evidence for the involvement of microtubules during Parkinson’s disease development and the potential therapeutic impact by regulating their dynamics have been discussed in a review paper by Pellegrini et al. (2017).

A summary of findings in the field of microtubule disorders in Parkinson’s disease and tauopathies is shown in Table 1. In conclusion, studies on various tauopathies confirmed that there is a strong interplay and positive feedback between pathogenic tau forms and dysfunctions of microtubules. Improving structure/function of the latter, exemplified by effects of paeoniflorin, might significantly improve functionality of affected neurons, thus, providing a possible way for attenuating the disease progress. On the other hand, due to complexity of pathomechanisms of Parkinson’s disease and remaining tauopathies, one should consider combined treatment procedures, targeting various pathological processes, to obtain positive therapeutic effects.

**Table 1:** Changes in microtubules in Parkinson’s disease and other tauopathies

Disease	Changes	Mechanism/consequence	Model	Reference
Parkinson’s disease	The presence of tubulin and microtubule associated proteins in Lewy’s Bodies	Lewy bodies contain tubulin which becomes unfunctional in such a form	Human	Galloway et al. (1988)
Parkinson’s disease	Appearance of specific inclusions affecting microtubules	Dysfunction of microtubules due to formation of inclusions as response to an increase in the level of abnormal proteins in neurons	Human	Puig et al. (2005)
Parkinson’s disease	Aggregation of gamma-tubulin; destabilization of microtubules, disruption of mitotic spindles and changes in the Golgi apparatus	Gamma-globulin aggregation in the centrosome promotes the formation of inclusions, and the compound itself, affecting the structure and function of the centrosome	Primary mesencephalic cell cultures, rats	Diaz-Corrales et al. (2005)
Parkinson’s disease	Aggregation of a synuclein and tubulin	The microtubule system is a potential target for a synuclein and alterations within it can lead to disorders	SH-SY5Y human neuroblastoma cells	Lee et al. (2006)
Parkinson’s disease	Changes in expression of genes coding for ATP synthase, β IIA- tubulin, and various enzymes	Increased susceptibility to the disease	Mice	Zempel et al. (2015)
Guam ALS/parkinsonism-dementia and Down syndrome	Neurofibrillary tangles appearance which affect microtubules	Involvement of tubulin in neurofibrillary tangle formation, which in turn cause destabilization of microtubules	Human	Schwab and McGeer (1998)
Frontotemporal dementia and Parkinsonism associated with chromosome 17	Changes in the cytoskeleton related to tau-mediated changes in the number and morphology of lysosomes	Lysosomal abnormalities may contribute to cytoskeleton dysfunctions and neurodegeneration	Mice	Lim et al. (2001)
Alzheimer’s disease and frontotemporal dementia and Parkinsonism associated with chromosome 17	Abnormal microtubule morphology	Modification of tubulin may lead to intermediate or late stages in the pathogenesis	Human	Boutté et al. (2005)
Frontotemporal dementia and Parkinsonism associated with chromosome 17 and related tauopathies	A specific mutation affects microtubule in a site- and isoform-dependent manner	Indication of the reason for the diverse clinical pathologies of FTDP-17 and related tauopathies	COS cells	Sahara et al. (2000)
Down syndrome, Alzheimer’s disease and Pick's disease	Reduced number of individual protein components in the brain affecting cytoskeleton	Identification of cytoskeleton elements in normal brain and indication of cytoskeleton defects in patients	Human	Pollak et al. (2003)
Alzheimer’s disease and Pick's disease	Abnormal hyperphosphorylated tau aggregates influence β II-tubulin	High β II-tubulin content as a factor contributing to the disease development	Human	Puig et al. (2005)
Undefined tauopathy	Reduced stability of detyrosinated microtubules	Aggregation of tau protein affects microtubules	Primary cultures of rat astrocytes	Yoshiyama et al. (2003)
Undefined tauopathy	Fragmentation of the Golgi apparatus	The mechanical strength of tau-induced ring microtubule bundles	SH-SY5Y human neuroblastoma cells	Rodríguez-Cruz et al. (2018)
Undefined tauopathy	Microtubule loss	The cytotoxic potential of the protein associated with microtubule-binding repeats	Transgenic models of *C. elegans*	Miyasaka et al. (2018)
Undefined tauopathy	A chaperone E knockout system of tubulin	Involvement of disruption of tubulin biosynthesis in the accumulation of toxic tau protein	primary cultures of mouse neurons	Fujiwara et al. (2018)
Undefined tauopathy	Abnormal aggregation of tubulin under the influence of glyceraldehyde	Elucidation of the mechanism of glyceraldehyde action in neurodegeneration which may contribute to the development of new neuroprotective therapies	SH-SY5Y human neuroblastoma cells	Nasu et al. (2020)
Undefined tauopathy	Abnormal microtubule binding, change in the distribution of filamentous actin	Indication of a newly discovered mechanism of tau protein toxicity in tauopathies	C6 glial cells	Maasz et al. (2014)

### Other Neurodegenerative Diseases

Extensive research on tubulin cytoskeleton dysfunctions has been carried out in the case of prion diseases caused by the accumulation of prion proteins (PrP). On the HeLa cell model with the inserted pcDNA3.1-PrP23-230 plasmid expressing human PrP23-230, the ability of PrP to bind to tubulin was indicated, which significantly influenced the continuity of the tubulin cytoskeleton (Li et al. 2011). The conducted studies showed not only the disruption of microtubule structures in cells accumulating cytosolic PrP and a decreased level of tubulin itself, but also a decreased viability of the cells and induction of apoptosis. The results of these studies were confirmed in the scrapie-infected hamster model in which reduced levels of endogenous tubulin in the nervous tissue were detected (Li et al. 2011). Other in vitro studies have also indicated an interaction between prion protein and tubulin polymerization promoting protein/p25 (TPPP/p25), known as a microtubule-associated protein (MAP) stabilizing microtubule fibers. The results of the tests carried out on scrapie-infected hamsters showed significant reductions in TPPP levels. In contrast, high levels of this protein in HeLa cell cultures prevented microtubule disruption and inhibited cell death caused by the accumulation of cytosolic PrP (Zhou et al. 2011). Zhang and Dong (2012) also collected data indicating decreased levels of MAP2 and excessively phosphorylated tau protein in many cellular models as well as in the brain tissues of naturally occurred and experimental human and animal prion diseases which correlates with decreased levels of tubulin (Zhang and Dong 2012). Studies on the HEK293 cell model also indicated the interaction of PrP with casein kinase 2 (the CK2 protein). Co-production of both subunits of this protein, CK2α and CK2β, restored the decreased level of tubulin and the disturbed structure of microtubules caused by the expression of the gene encoding the prion protein (Wang et al. 2013). Another kinase, microtubule affinity-regulating kinase 4 (MARK4), also turned out to be one of the proteins whose levels were decreased in prion diseases. Gong et al. proved that MARK4 levels in scrap 263 K-infected hamsters, 139A-infected mice and a case of Creutzfeldt-Jakob disease characterized by significant PrP deposits were almost undetectable (Gong et al. 2012). These data clearly indicated that PrP, by interacting with tubulin itself or proteins affecting the stabilization of the tubulin cytoskeleton, causes a decrease in the level of tubulin and disruption of the continuity of the cytoskeleton which has huge consequences, mainly for nerve cells. On the other hand, studies in mice lacking the gene encoding the cellular prion protein PrP (Prnp^0/0^) showed a reduced number of β tubulin III-positive neurons and pyramidal cells in the dentate gyrus, indicating that PrP^C^ must play a significant role in the maintenance of the microtubule composition (Schmitz et al. 2014). Interestingly, post-translational modifications of *α*-tubulin was considerably altered under condition of the PrP^C^ depletion (Halliez et al. 2015) which is an important discovery in the light of the recently demonstrated crucial role of tubulin polyglutamylation control in cellular homeostasis, transportation and prevention of neurodegeneration (Bodakuntla et al. 2021a). Impaired intracellular transport, arising from decreased efficiency of α-tubulin ubiquitination, was also observed in cells with mis-localized PrP (Mukherjee et al. 2017). Moreover, β tubulin was among 15 proteins demonstrated to interact specifically with PrP (Zafar et al. 2017). It is also worth to stress that the “prion hypothesis” has been proposed, in which unproper folding of some natural proteins (like Aβ or tau), and appearance of untypical forms revealing pathogenic properties, can facilitate or even force misfolding of other proteins of similar primary structure that are abundant in the brain; this might trigger the pathogenic cascade leading to neurodegeneration (Han et al. 2022; Lukiw 2022).

Changes in the level of the aforementioned MAP2 protein have also been observed in Huntington's disease. In brain specimens from patient-derived frontal cortex and striatum, splicing disorders were noted, indicating the advantage of mRNA of low molecular weight MAP2 isoforms (LMW-MAP2) over the almost undetectable high molecular weight isoforms (HMW-MAP2) (Cabrera et al. 2017). This was accompanied by a decrease in the total level of MAP2 protein. It was suggested that the mechanism of these disorders indicate the SR splicing factor (SRSF6) which may initiate such changes (Cabrera et al. 2017). Other studies conducted on frontal cortex of patients showed a decrease in the levels of markers of the normal cytoskeleton organization a-tubulin, microtubule-associated protein 2 (MAP2), and phosphorylated neurofilament (DiProspero et al. 2004). It was concluded that a mutant form of huntingtin, by interacting with these proteins, affects the integrity of the tubulin cytoskeleton before the first symptoms of the disease appear (DiProspero et al. 2004). Interesting data pointing to Huntington's disease-related cytoskeletal abnormalities in a mouse model indicated the presence of hyperacetylated a-tubulin in the cilia photoreceptor, possibly related to the accumulation of the mutant form of huntingtin (Karam et al. 2015). The consequence of the aforementioned phenomenon is a disturbance of the specific transport function, aberrant accumulation of outer segment proteins in the photoreceptor cell bodies and disruption of outer segment integrity and finally cell death (Karam et al. 2015).

Letournel et al. (2003) reported interactions between abnormally accumulated neurofilaments from toxic axonal spheroids and stable tubule only polypeptide (STOP) responsible for stabilizing microtubules, the level of which increases significantly in the spinal cords and brains of patients with amyotrophic lateral sclerosis. Moreover, other microtubule proteins, such as tubulin and kinesin, were present in the spheroids, indicating that the microtubule destabilization process may be involved in the pathogenesis of this disease (Letournel et al. 2003). Previous studies also indicated modifications of the β-tubulin group and abnormal protein spots that seemed to be closely related to tau proteins in intercostal nerves from amyotrophic lateral sclerosis patients (Binet and Meininger 1988).

Changes in tubulin cytoskeleton in prion diseases, Huntington’s disease, and amyotrophic lateral sclerosis are summarized in Table 2. In conclusion, not only P-tau, but also other pathological proteins, like pathological PrP or mutant huntingtin, can interact with tubulin and significantly affect structure and functions of microtubules. On might suggest that preventing such interactions and improving the tubulin cytoskeleton might be important in effective treatment of patients suffering from the discussed group of diseases. In fact, treatment of cellular model of Huntington’s disease, as well as cells derived from patients with this disease, with a compound stimulating autophagy, corrected the phenotypes of cells (Pierzynowska et al. 2018, 2019b), analogously to results obtained with a model of the Alzheimer’s disease (Pierzynowska et al. 2019a). The mechanism of action of this potential therapy is most probably degradation of various protein aggregates which also prevents dysfunctions of the cytoskeleton.

**Table 2:** Changes in tubulin cystoskeleton in prion diseases, Huntington’s disease, and amyotrophic lateral sclerosis (ALS)

Disease	Cytoskeleton change	Mechanism of changes	Model	Reference
Prion disease	Disruption of microtubule structures; decreased level of tubulin	Interaction between PrP and tubulin	HeLa cell expressing human PrP23-230	Li et al. (2011)
		Interaction between PrP and CK2 protein	HEK293 cells transfected with various PrP constructs	Wang et al. (2013)
	Reduced levels of endogenous tubulin	–	Scrapie-infected hamsters	Li et al. (2011)
	Disruption of microtubule structures	Reductions in TPPP levels	Scrapie-infected hamsters	Zhou et al. (2011)
		Reductions in MARK4 levels	Scrapie-infected hamsters; 139A-infected mice	Gong et al. (2012)
Huntington disease	Disruption of microtubule structures	Reduction of MAP2 levels	Brain specimens from patients	Cabrera et al. (2017)
	Disruption of microtubule structures and decreased level of tubulin	Reductions of levels of MAP2 and phosphorylated neurofilament	Brain specimens from patients	DiProspero et al. (2004)
	Presence of hyperacetylated α-tubulin	–	R6/2 transgenic mice	Karam et al. (2015)
Amyotrophic lateral sclerosis	Disruption of microtubule structures	Interaction between stable tubule only polypeptide (STOP) and accumulated neurofilaments	Brain specimens from patients	Letournel et al. (2003)
	Modifications of the β-tubulin group	–	Intercostal nerves from patients	Binet and Meininger (1988)

A general mechanism which can lead to changes in the cytoskeleton, including but not limited to microtubules, has been reported recently in mucopolysaccharidoses (MPS), a group of lysosomal storage diseases caused by dysfunctions of enzymes involved in degradation of glycosaminoglycans (GAGs). In cells of the patients, GAGs accumulate causing direct dysfunctions of lysosomes in a progressive manner which leads to severe symptoms, including neurodegeneration (for a recent review on pathomechanisms and diagnosis of MPS, see Wiśniewska et al. 2022). Apart from the primary biochemical defect, changes in many organelles have been observed as indirect effects of GAG storage, apparently due to significant changes in expression of hundreds of genes in MPS cells (Gaffke et al. 2020). Among them, several dozens of genes coding to proteins related to cytoskeleton were dysregulated in patients’-derived cells, with those involved in the microtubule organizing center being in the group of the most severely affected ones (Gaffke et al. 2021). Interestingly, one of the dysregulated genes in MPS cells was *PLXNA1*, encoding plexin, a protein important for functions of the cytoskeleton (Rintz et al. 2020). Therefore, one can propose that since changed levels of plexin may result in improper development of CNS and its dysfunction (Meyer et al. 2016), neurodegenerative processes observed in many MPS types could be related (at least partially) to dysregulation of the *PLXNA1* gene and subsequent cytoskeleton disorders. In this light, it will be important to test whether expression of genes coding for proteins related to structures and functions of the cytoskeleton, and especially microtubules, is dysregulated also in other neurodegenerative diseases.

## Microtubule Alterations in Glial Cells

The majority of the discussions presented above concerned dysfunctions of tubulin cytoskeleton in neurons. However, the question appears if similar changes occur also in glial cells and if they can contribute to neurodegeneration. In fact, one of major dysfunctions leading to microtubule alterations other than primary tubulinopathies – tau-related disorders (see Fig. 1 for a summary) – were described to occur in both neurons and glial cells (Leyns and Holtzman 2017). Despite the fact that in the brain the tau protein is synthesized predominantly neurons, it was reported to be present also in oligodendrocytes and astrocytes, though at lower levels (Kahlson and Colodner 2016). Nevertheless this is sufficient to cause problems with tubulin cytoskeleton if tauopathy occurs (Odfalk et al. 2022). Early studies, summarized by Richter-Landsberg (2008), demonstrated the importance of the cytoskeleton, and especially microtubules, in functions of oligodendrocytes, as well as the deleterious effects of dysfunctions of these cellular structures. As mentioned in one of preceding subsections (“Parkinson’s disease and remaining taoupathies”), a tau aggregate-mediated decrease in stability of microtubules in rat astrocytes caused dysfunctions and subsequent death of these cells (Yoshiyama et al. 2003; Kahlson et al. 2016). Moreover, defects in microtubule binding by a pathologically shortened tau form was reported in glial cells (Torres-Cruz et al. 2016).

More recent works confirmed the conclusions on importance of microtubule changes in glial cells to various brain disorders. For example, alterations in contents of astrocytic proteins GFAP and TSP-1 correlated with dysfunctions of tubulin cytoskeleton and neuronopathy in model C58/J mice (Barón-Mendoza et al. 2018). Microtubules occurring in oligodendrocytes are classified as radial and lamellar types (Crockett 2021). When the gene coding for the tubulin polymerization promoting protein was mutated in the mouse knock-out model, oligodendrocytes had shorter lamellar microtubules which corelated with formation of short and thin myelin sheaths, as well as with altered behavior (Fu et al. 2019). In the mouse model of the Rett syndrome (a neurodegenerative disease), a specific deficit in microtubule dynamics was detected in astrocytes (Lebrun et al. 2021).

The above presented facts indicate that although studies on microtubule alterations in glial cells were less frequent than those with analyses of neurons, dysfunctions of tubulin cytoskeleton in oligodendrocytes and astrocytes were correlated with neurodegenerative processes. The most probable mechanisms by which perturbations in microtubule structure and dynamics affect the brain function seem to be related to impaired myelinization and dysregulation of the immune response in CNS. Definitely, more studies on changes in microtubules in glial cells during neurodegeneration are necessary to understand these mechanisms in more detail.

## Concluding Remarks

**Fig. 3:**
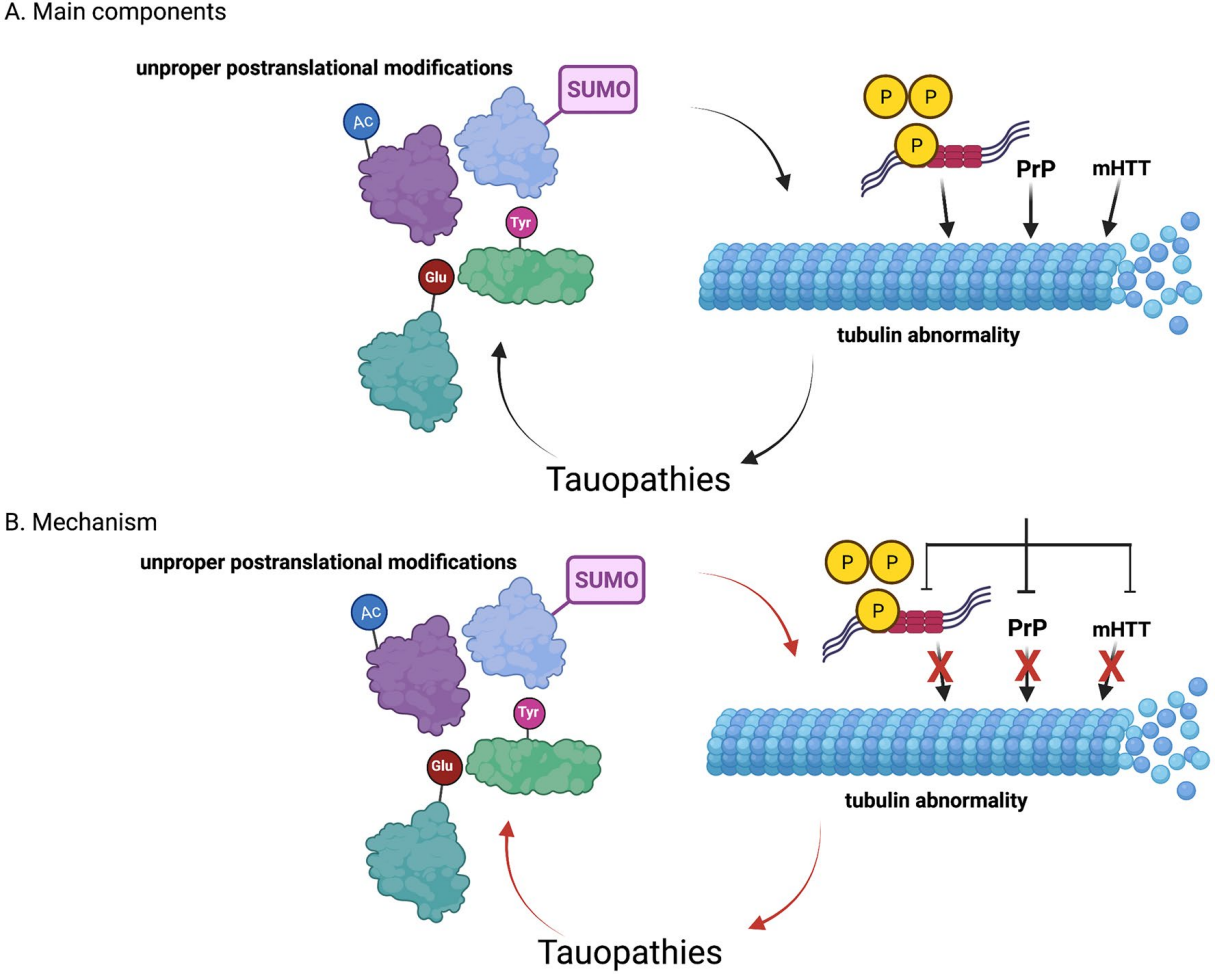
The main components of the pathogenic spiral influencing microtubules in various neurodegenerative diseases (**A**), and potential therapeutical approaches disrupting such a spiral and recovering cytoskeleton homeostasis (**B**). Hyperphosphorylated tau protein (P-tau), prion protein (PrP), or mutant huntingtin (mHTT) can cause tubulin abnormality which, in turn, enhances tauopathy. This process in further stimulated by unproper post-translational modifications of tubulin (tyrosination, acetylation, polyglutamylation, and SUMOylation appear to be the most important tubulin modifications). Therefore, the pathogenic spiral is established (**A**). To disrupt this spiral and to recover cytoskeleton homeostasis, removal of P-tau, PrP, or mHTT can be proposed, for example by stimulation of the autophagy process (**B**). Figures were created using BioRender.com

Dysfunctions of tubulin cytoskeleton are often observed in various neurodegenerative diseases. These can be either primary neurodegenerative tubulinopathies (caused by mutations in genes coding for components of microtubules, like *TUBA4A*, *TUBB2A*, and *TUBB3*) or disorders caused by various pathogenic interactions and transactions between different compounds and tubulin cytoskeleton. Such dysfunctions are observed especially in Alzheimer’s disease, Parkinson’s disease, Huntington’s disease, amyotrophic lateral sclerosis, and prion diseases. Dysregulation of genes coding for proteins involved in the cytoskeleton structure and functions has been reported in MPS. Importantly, during neurodegeneration, changes in microtubules were reported in both neurons and glial cells. Understanding principles of molecular mechanisms of microtubule disorders in these diseases leads to the proposal that their correction might attenuate the disease progress. As a result, finding newly recognized molecular targets for potential drugs might become possible in near future. Of special interest are the microtubule defects caused by either unproper post-translational modifications of tubulin molecules which result in defective intracellular transportation or P-tau and other pathogenic proteins, like toxic forms of PrP and huntingtin, which in turn enhance tauopathy. Interruption of such a pathogenic spiral might be an attractive therapeutic option, and recent experiments on cellular and animal models of Huntington’s and Alzheimer’s diseases suggested that such an approach may be effective in correction of phenotypes of affected cells and organisms. Main components of the pathogenic spiral in various neurodegenerative diseases are shown in Fig. 3A, and proposed therapeutical targets are indicated in Fig. 3B.

## Data Availability

Enquiries about data availability should be directed to the authors.
